# Machine learning model for depression based on heavy metals among aging people: A study with National Health and Nutrition Examination Survey 2017–2018

**DOI:** 10.3389/fpubh.2022.939758

**Published:** 2022-08-04

**Authors:** Fang Xia, Qingwen Li, Xin Luo, Jinyi Wu

**Affiliations:** Department of Public Health, Wuhan Fourth Hospital, Wuhan, China

**Keywords:** depression, metal elements, machine learning, online application, aging

## Abstract

**Objective:**

To explore the association between depression and blood metal elements, we conducted this machine learning model fitting research.

**Methods:**

Datasets from the National Health and Nutrition Examination Survey (NHANES) in 2017–2018 were downloaded (https://www.cdc.gov/nchs/nhanes). After screening, 3,247 aging samples with 10 different metals [lead (Pb), mercury (Hg), cadmium (Cd), manganese (Mn), selenium (Se), chromium (Cr), cobalt (Co), inorganic mercury (InHg), methylmercury (MeHg) and ethyl mercury (EtHg)] were included. Eight machine learning algorithms were compared for analyzing metal and depression. After comparison, XGBoost showed optimal effects. Poisson regression and XGBoost model (a kind of decision tree algorithm) were conducted to find the risk factors and prediction for depression.

**Results:**

A total of 344 individuals out of 3247 participants were diagnosed with depression. In the Poisson model, we found Cd (β = 0.22, *P* = 0.00000941), EtHg (β = 3.43, *P* = 0.003216), and Hg (β=-0.15, *P* = 0.001524) were related with depression. XGBoost model was the suitable algorithm for the evaluation of depression, the accuracy was 0.89 with 95%CI (0.87, 0.92) and Kappa value was 0.006. Area under the curve (AUC) was 0.88. After that, an online XGBoost application for depression prediction was developed.

**Conclusion:**

Blood heavy metals, especially Cd, EtHg, and Hg were significantly associated with depression and the prediction of depression was imperative.

## Introduction

Depression was characterized by significant persistent upset. Depression is an increasingly serious global mental health problem, resulting in a significant decline in physical function and quality of life but an increase in disease incidence and mortality. In 2017, about 17.3 million adults aged 18 and over in the United States experienced at least one episode of major depression, with a prevalence of about 7.1% (1). According to the “depression and other common mental diseases: global health estimates” released by world health organization (WHO) in 2017, the total number of patients with depression in the world was 322 million. Nearly half of them live in Southeast Asia and the Western Pacific, such as China and India. In China, depressive disorders have been estimated to be the second leading cause of years lived with disability (2). At the same time, the prevalence of depression varies with age and reaches a peak in the elderly. It is estimated that the prevalence of depression among women aged 55–74 was more than 7.5%. In 2015, depression led to more than 50 million years of disability (YLD) worldwide, of which 80% occurred in low-income and middle-income countries (3). Depression is characterized by a lack of vitality, sadness, insomnia, and an inability to enjoy life. The world depression survey conducted in 17 countries found that on average, 1 in 20 people had depression. By 2020, the global disease burden might increase by 5.7%, becoming the second largest disease after ischemic heart disease (4).

Due to invalid treatment and low cure rate, identifying the risk factors for depression was imperative. Early prevention and intervention could effectively delay the progress of depression. Several factors were reported to be associated with depression, including age, gender, work, and lifestyle (5). Recently, the association between environmental pollutants and depression has also received a lot of attention. It is well documented that exposure to a wide range of chemicals, especially heavy metals could lead to depression. Heavy metals had been regarded as environmental risk factors for several health damages, including lung function, cardiovascular diseases, and diabetes (6). However, evidence for the association between heavy metals and depression was limited. Only one study explored the association (7), and in the research they just analyzed the association of Pb, Hg, Cd, and depression using a cross-sectional study. The risk prediction of depression with heavy metal exposure was unknown.

Machine learning (ML) is a subset of artificial intelligence (AI), which focuses on teaching computers to learn from data and achieve empirical improvement through explicit programming. In machine learning, after training, the algorithm can find data patterns and correlations in large data sets, and make the best decision and prediction (8). Machine learning applications get improved as they are used, and the more data they can access, the more accurate they are. Machine learning applications are now everywhere: our homes, shopping carts, entertainment media, and health care (9). One of the common uses of machine learning is classification, which can be used to predict the target events based on the risk factors. The assessment of risk factors not only depends on the ML algorithm but also on the medical significance. It is reported that ML has better performance than logistic regression in clinical research since ML has a better customized process than logistic regression.

Therefore, we conducted this study to examine the association between heavy metal exposure and depression and to explore the risk factors for depression. Meanwhile, eight machine learning algorithms were compared for model training and testing. The prediction model of heavy metals exposure to depression was realized, and a relevant online application was developed.

## Methods

### Dataset

In this research, the National Health and Nutrition Examination Survey (NHANES) in 2017–2018 was used for machine learning model fitting and prediction for depression associated with 10 kinds of heavy metals. NHANES was a program of studies designed to assess the health and nutritional status of adults and children in the United States. The NHANES questionnaires included demographic, socioeconomic, dietary, and health-related questions. The examination component consists of medical, dental, and physiological measurements, as well as laboratory tests administered by highly trained medical personnel. All the data could be accessed on the website of the American Centers for Disease Control and Prevention (https://www.cdc.gov/nchs/nhanes).

To analyze the association between depressive symptoms and 10 kinds of heavy metals, we extracted the demographic data, blood heavy metal data in the laboratory, and data from a depression level questionnaire. The demographic data included several health-related factors. Blood heavy metal include Pb, Cd, Hg, Se, Mn, InHg: EtHg, MeHg, Cr, Co. The depression results included ten questions with no depression, which could be calculated as the final result of depression.

**Figure 1:**
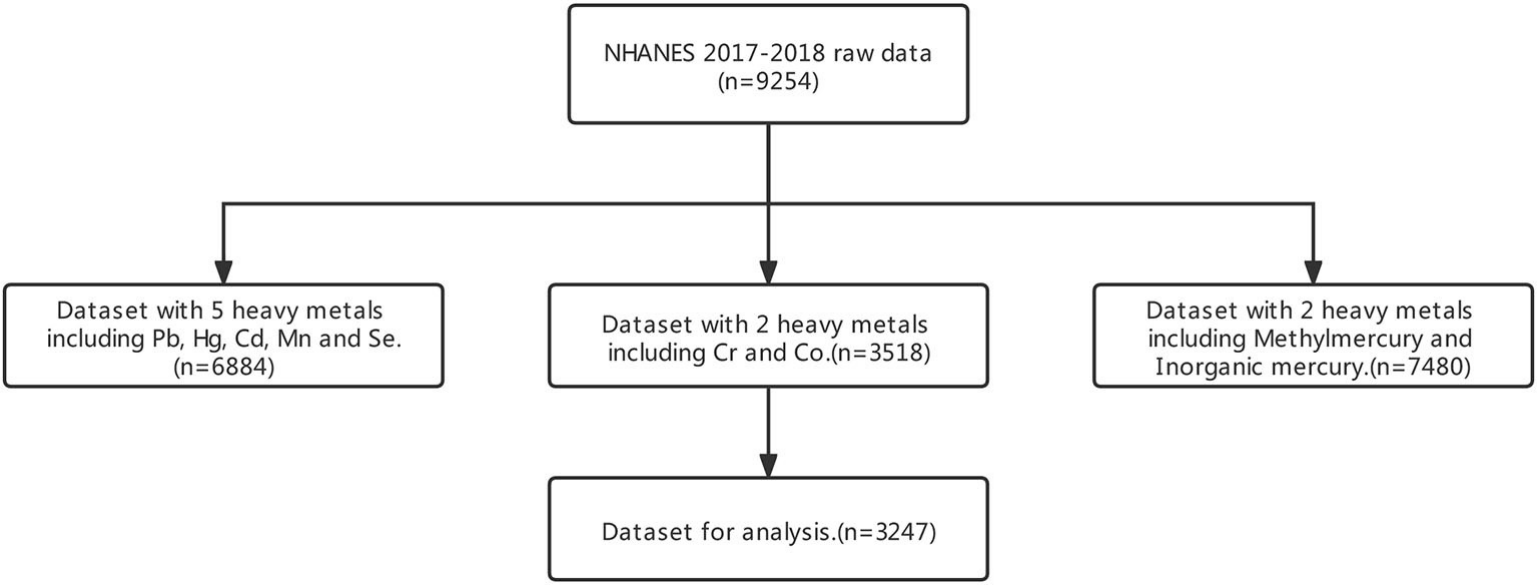
Flowchart of dataset combination.

Since some samples lack InHg or EtHg information, not all participants from 2017 to 2018 were selected for the research. In the raw data, there were original 9254 participants. After screening, only 3,247 respondents have lab data and depression questionnaire data. The selection process of participants is shown in Figure 1. In the data processing, to guarantee the data quality, we delete all missing values in the needed variables and merge sub-datasets to be analyzed into one table based on a unique identifier. Since the official data quality department had to check the whole dataset, we kept the original data as much as possible, and only deleted the records with missing values.

### Evaluation of depression

With the introduction of NHANES official documents, a nine-item depression screening instrument, also called the Patient Health Questionnaire (PHQ-9) was administered to determine the frequency of depression symptoms over the past 2 weeks (10). The PHQ-9 has been proven to be a valid tool with high specificity and sensitivity in evaluating symptoms of depression (11).

This screening instrument incorporates DSM-IV depression diagnostic criteria. The PHQ-9 consists of 10 questionnaires, which are based on the diagnostic criteria of depression from the Diagnostic and Statistical Manual of Mental Disorders IV (DSM-IV) (11). It is a reliable and valid measurement in screening for depression. For those who have complete answers to a nine-item depression screening instrument (PDQ), the total score can be calculated, ranging from 0 to 27. Major depression and depression severity can be assessed using predefined cut points. For each participant, a total score of ten questions on depression was the criteria. The criteria were that the total score ≥10, and the participant would be considered as depressed if the total score was equal to or more than 10 (12).

### Assessment of heavy metals

Blood heavy metals were directly measured in Pb, Cd, Hg, Mn, and Se content of whole blood specimens using mass spectrometry after a simple dilution sample preparation step. During the sample dilution step, a small volume of whole blood was extracted from a larger whole blood patient specimen after the entire specimen was mixed to create a uniform distribution of cellular components. The dilution of the blood in the sample preparation step prior to analysis was a simple dilution of 1 part sample + 1 part water + 48 parts diluent. Liquid samples were introduced into the mass spectrometer through the inductively coupled plasma (ICP) ionization source.

The quantification of InHg, MeHg, and EtHg in whole blood samples was performed using a triple spike isotope dilution (TSID) method employing gas chromatography (GC) to separate the species followed by introduction into an ICP-DRC-MS for detection. TSID was a specialized extension of the Isotope Dilution technique. TSID measured individual chemical species (inorganic, methyl, and ethyl mercury species) in samples using ID principles. The blood sample was spiked with known amounts of each Hg species that had been enriched with isotopic variants of the target element of interest.

The concentrations of chromium (52Cr) and cobalt (59Co) in whole blood specimens were directly measured using inductively coupled plasma mass spectrometry (ICP-MS). This analytical technique was based on analyzing detection using quadrupole ICP-MS technology, including Kinetic Energy Discrimination (KED) technology, which minimizes or eliminates many argon-based polyatomic interferences.

### Statistical analysis

In the baseline data analysis, Chi-square test and *t*-test were used to analyze the difference between demographic data and heavy metals in two groups.

Subsequently, we used the Pearson correlation matrix and Poisson regression to identify meaningful heavy metals associated with depression, since Poisson regression was the most common model for fitting count data. After that, we compared 8 machine learning algorithms based on accuracy and kappa value. The best machine learning model would be used to make the prediction for depression levels based on meaningful heavy metals. To validate the prediction effects, cross-validation was conducted, in which 80% of the data was used to train the model and 20% of the data was used to make the prediction testing. Then, the prediction value would be evaluated with 20% original data. The confusion matrix and ROC curve would be used to evaluate the prediction effects. All the analyses were conducted in R software 4.1.2 (The R Foundation for Statistical Computing, USA). Two-sided *P* < 0.001 was considered statistically significant.

### Machine learning component

In our study, we compared 8 different machine learning algorithms, including Extreme Gradient Boosting (XGBoost), Decision Tree (DT), Support Vector Machine (SVM), Multivariate Adaptive Regression Splines (MARS), Artificial Neural Networks (ANN), Boosted Trees (BT), Random Forest (RF), K-Nearest Neighbors (KNN). All 8 algorithms were conducted in R software. To achieve the best performance of each algorithm, we tuned the hyperparameters of each algorithm.

Since the XGBoost algorithm predicted depression best, it was described without detailing the other seven algorithms (13, 14). The XGBoost algorithm library offers high efficiency, flexibility, and portability while being optimized and distributed. In this framework, gradient boosting is used for implementing machine learning algorithms. Gradient Boosting Decision Tree (also known as GBM, GBDT) is a data science technique that solves many problems quickly and accurately. Using the same code, we could solve problems beyond billions of examples in major distributed environments (Hadoop, SGE, MPI) (13, 14).

The principle of the XGBoost algorithm could be summarized as follows:

Assumed a training dataset *D* = {( *x*_*i*_ , *y*_*i*_), *i* = 1..*n*} of the size *n*, where *x*_*i*_= (*x*_*i*__1_ , *x*_*i*__2_ ,…, *x*_ℑ_) denoted an *m*-dimensional feature vector with the corresponding (output) category *y*_*i*_:


(1) $$\begin{array}{r} {\hat{y}}_{i}\begin{array}{c} ={\sum^{\text{​}}}_{k=1}^{k}f_{k}(x_{i}),f_{k}\in F \end{array} \end{array}$$

Where, *K* represented the number of trees, *f*_*k*_ (*x*_*i*_) represented the score that is associated with the model's *k*th tree, and *F* denoted the space of scoring functions available for all boosting trees.

Different from another tree-based algorithm GBDT (gradient boosting decision tree), in the XGBoost algorithm, a regularization term was added to the objective function to avoid the overfitting problem (7).

### XGBoost model fitting and validation

First, we divided the original data into training data and testing data according to 8:2, which means that 80% of the original dataset was used for training the model and 20% of that was for model testing. The training data was used to train the XGBoost model, and the testing data was used to verify the model. We validated all the algorithms through 10-fold cross-validation.

In the model fitting process, we set the parameters, including the number of boosting trees, max depth of trees, eta, rate of drop, skip of drop, colsample bytree, minimal child weight, subsample, and gamma. The values of parameters are as follows: *n*rounds = *c*(1,10,20), max_depth = 2, eta = 0.1, rate_drop = 0.10, skip_drop = 0.10, colsample_bytree = 0.90, min_child_weight = 2, subsample = 0.75, gamma = 0.10. Usually we should set a group of hyperparameters like eta = *c*(0.01, 0.001, 0.0001), max_depth = *c*(2,4,6,8,10) and produce plot to find the tune the best parameters. In the application, we already input the best hyperparameters.

To select features and evaluate the model fitting, a variable importance plot and ROC curve were conducted. In addition, a confusion matrix was applied to evaluate the prediction of the XGBoost model and several indicators were used, including sensitivity, specificity, positive prediction value, negative prediction value, Kappa value, and accuracy.

## Results

On average, the sample was comprised of the elderly (mean ± SD; 60.8 ± 11.9 years old), predominantly married (*n* = 1827, 56.3%), four races (White, Black, Mexican, Hispanic), male (*n* = 1601, 50.7%) and female (*n* = 1646, 49.3%), some college (*n* = 1023, 31.5%) and college graduate (*n* = 777, 23.9%), married (*n* = 1827, 56.3%), overweight (*n* = 1433, 44.1%) and obese(*n* = 1108, 34.1%), PIR 1.0–4.0 (*n* = 3247, 55.77%), lower socio-economic (*n* = 2228, 68.62%), drinking (*n* = 2915, 89.8%) but not smoking (*n* = 1762, 54.3%) (Table 1). In the differences analysis of the baseline data, only gender and race showed no significant difference between two status groups. Furthermore, we list the heavy metals from the samples' blood testing. All the units of heavy metals were ug/L. *T*-tests of all the metals showed that blood cadmium, blood mercury, and blood methylmercury had significant differences among the four depression statuses (Table 2).

**Table 1:** Demographic of adults aged 40 years or older by depression in NHANES 2017–2018 (*n* = 3,247).

**Items**	**Depression status**	**No(2903)**	**Yes(344)**	**Chi-square test and *t*-test**
**Gender**	Female	1436	210	χ2 = 2018.5^*^
	Male	1467	134	
**Age**	Age	60.63 ± 11.74	60.06 ± 11.27	t = 0.84
**Race**	Non-Hispanic Black	676	74	χ2 = 7.5
	Other race–including multi-racial	529	45	
	Non-Hispanic White	1067	142	
	Mexican American	363	48	
	Other Hispanic	268	35	
**Education**	Don't know-11th grade	305	51	χ2 = 43.96^*^
	Less than don't knowth grade	258	45	
	Some college	911	112	
	High school graduate	688	94	
	College graduate	737	40	
	Refused	2	0	
	Don't know	2	2	
**Marriage**	Never married	231	37	χ2 = 54.8^*^
	Living with partner	163	20	
	Married	1695	132	
	Divorced	410	79	
	Widowed	291	55	
	Separated	109	20	
	Refused	4	1	
**BMI**	<18.5 (underweight)	27	4	χ2 = 35.43^*^
	18.5–24.9 (normal)	624	51	
	25–29.9 (overweight)	1022	86	
	≥30 (obese)	1230	203	
**PIR**	≥4.0	914	55	χ2 = 54.12^*^
	1.0–4.0	1607	204	
	<1.0	382	85	
**Occupation**	upper socialeconomic	926	47	χ2 = 49.65^*^
	lower socialeconomic	1939	289	
	unemployed	38	8	
**Drinking status**	No	310	24	χ2 = 4.57^*^
	Yes	2593	320	
**Smoking status**	No	1612	150	χ2 = 17.62^*^
	Yes	1291	194	

* P < 0.05.

**Table 2:** Environmental heavy metals of adults aged 40 years or older by depression in NHANES 2017–2018.

**Depression**	**No(2903)**	**Yes(344)**	***t*-test**
Blood lead(ug/L)	1.46 ± 1.56	1.31 ± 1.03	t = 2.36
Blood cadmium(ug/L)	0.5 ± 0.57	0.65 ± 0.73	t = 12.16^*^
Blood mercury(ug/L)	1.6 ± 2.87	1.03 ± 1.68	t = 5.19^*^
Blood selenium(ug/L)	191.84 ± 28.2	189.41 ± 24.4	t = 2.54
Blood manganese(ug/L)	9.71 ± 3.5	9.7 ± 3.56	t = 0.95
Blood inorganic mercury(ug/L)	0.25 ± 0.62	0.2 ± 0.15	t = 0.75
Blood ethylmercury(ug/L)	0.05 ± 0.01	0.05 ± 0.05	t = 1.57
Blood methylmercury(ug/L)	1.33 ± 2.54	0.83 ± 1.53	t = 5.26^*^
Blood chromium(ug/L)	0.36 ± 0.34	0.32 ± 0.13	t = 1.39
Blood cobalt(ug/L)	0.52 ± 0.2	0.2 ± 0.17	t = 2.55

* *P < 0.001*.

**Figure 2:**
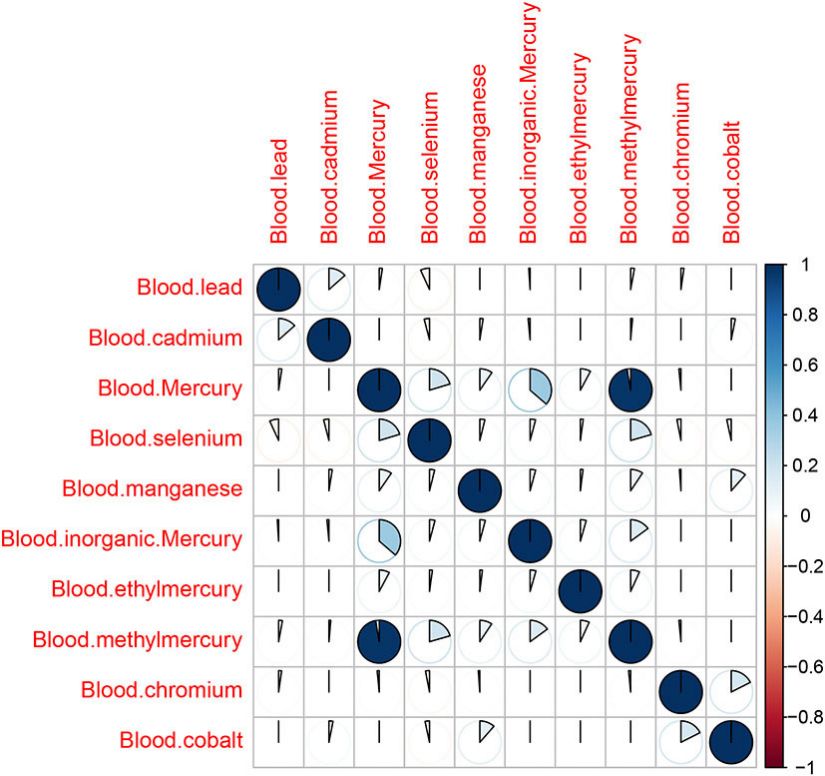
Correlation matrix among 10 metal elements.

First, Pearson correlation matrix analysis was conducted for the correlation of 10 environmental heavy metals (Figure 2). According to the matrix, there was an obvious correlation between methylmercury and mercury (the correlation value is 0.91, *P* < 0.001). Therefore, methylmercury should be eliminated in the next Poisson review. Exploratory factor analysis confirmed high correlated covariant and the other 9 metals had limited correlation with each other.

To further analyze the risk level of 9 heavy metals, we conducted the Poisson regression (since the dependent variable depression status corresponds to Poisson distribution) (Table 3). The significant heavy metals related to depression were blood Cd (estimate = 0.22, *P* = 0.00001), blood Hg (estimate = −0.15, *P* = 0.002), and blood EtHg (estimate = 3.43, *P* = 0.003), respectively. This model's results showed that cadmium and ethyl mercury may be harmful to depression while mercury may be helpful for depression patients.

**Table 3:** Poisson regression model of 9 metal elements.

**Model fit**	**β**	**Std. Error**	***z*–value**	***P*–value**
(Intercept)	−1.61	0.46	−3.55	0.0004^*^
Blood.lead	−0.11	0.05	−1.97	0.06
Blood.cadmium	0.22	0.05	4.43	0.00001^*^
Blood.mercury	−0.15	0.05	−3.17	0.002^*^
Blood.selenium	−0.002	0.002	−0.79	0.42
Blood.manganese	0.006	0.016	0.38	0.71
Blood.inorganic.mercury	−0.41	0.44	−0.94	0.35
Blood.ethylmercury	3.43	1.16	2.95	0.003^*^
Blood.chromium	−0.76	0.40	−1.88	0.06
Blood.cobalt	−0.05	0.15	−0.31	0.76
**Model evaluation**
AIC	2203.6			

* P < 0.001.

**Figure 3:**
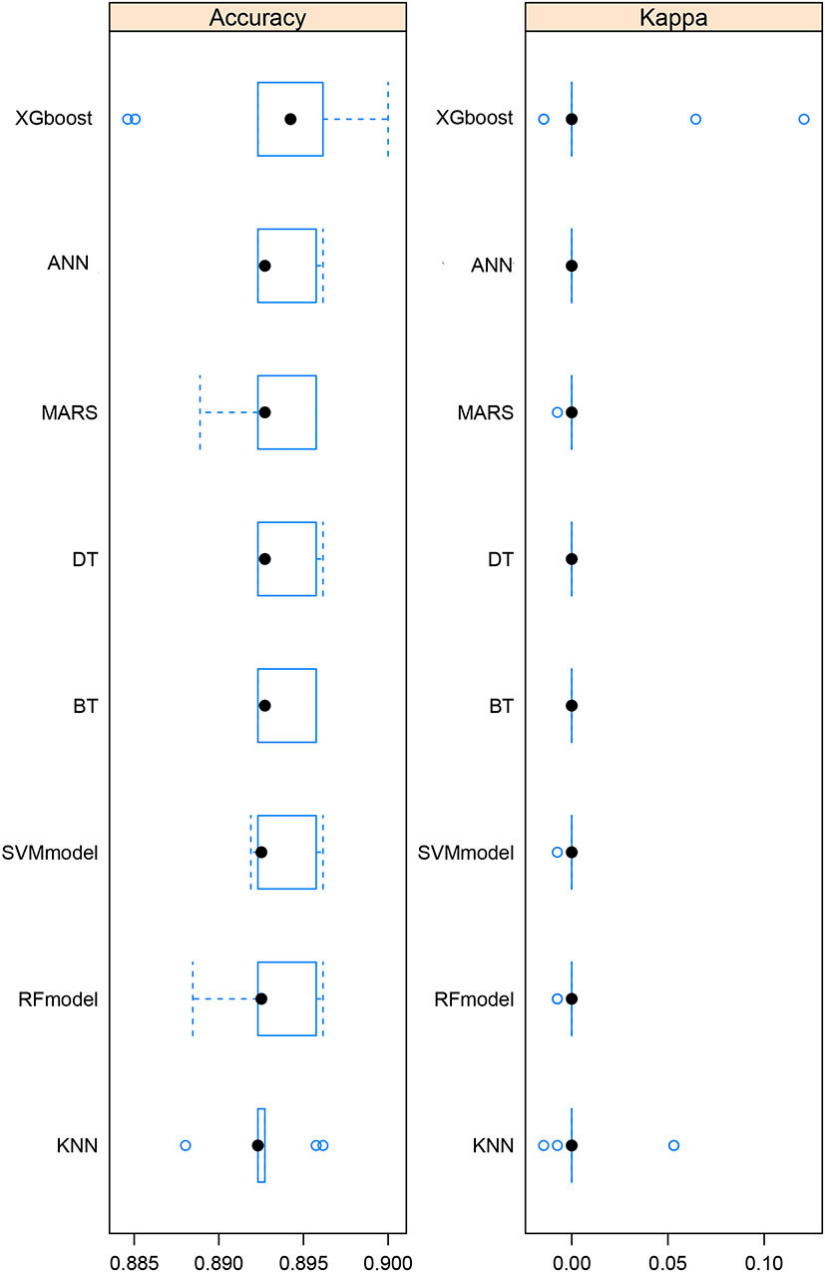
Comparison among 8 machine learning algorithms. The accuracy and kappa value of each algorithm are shown.

After the exploratory analysis, we made 8 kinds of machine learning model fit (Figure 3). Then, we evaluated the accuracy of each model by counting how many of the predicted record numbers matched the actual record numbers in the testing data, and we conducted a hypothesis test under the null hypothesis that the number of matches follows a binomial distribution with a success probability corresponding to the inverse of the number of individuals. This was a test of the null hypothesis that all matches were attributable to pure chance, and it was a test of reidentification because we were quantifying the ability of various models computed by machine learning algorithms to match activity data to unique identifiers. The comparison between 8 algorithms was based on the accuracy of prediction and kappa value (this value represents the agreement, which ranges from −1 to +1. The higher the value of kappa, the stronger the agreement). The AUC values were calculated for the other 7 models (DT AUC = 0.80, SVM AUC = 0.77, MARS AUC = 0.74, ANN AUC = 0.75, BT AUC = 0.75, RF AUC = 0.82, and KNN AUC = 0.76). From the comparison, we found that the XGBoost model was the best choice for fitting the relation between depression status and heavy metals.

The XGBoost result also produced the variable importance plot of 9 heavy metals after the screening by the Poisson model (Supplementary Figure 1), and it demonstrated that cadmium, mercury, and lead take the first to third places in the importance rank while chromium takes the second to last. In addition, a receiver operating characteristic (ROC) curve was made to evaluate the XGBoost prediction efficacy, which showed that the area under the curve (AUC) was 0.88, and which was a good model fitting Supplementary Figure 2.

To show the prediction efficacy of XGBoost in more detail, we conducted the confusion matrix (Table 4). When creating a confusion matrix, we include both predictive and actual values you test in the system. Each row should correspond to each predicted class, and each column corresponds to the actual class. The overall accuracy of the model was 0.89 with a 95%CI (0.87, 0.92) and Kappa value was 0.006. The statistics by class of the model included sensitivity, specificity, positive prediction value, and negative prediction value, which showed the good accuracy of the model prediction.

**Table 4:** Confusion matrix of XGBoost model.

**Prediction**
	**no**	**yes**
**No**	570	11
**Yes**	10	57
**Overall statistics**
Accuracy	0.89	
95%CI	(0.87, 0.92)	
Kappa	0.023	
**Statistics**
Sensitivity	0.98	
Specificity	0.84	
Positive predicted value	0.98	
Negative predicted value	0.85	
Prevalence	0.89	
Detection rate	0.88	
Detection prevalence	0.89	
Balanced accuracy	0.91	

To further promote the application of the research, we also developed the dynamic interactive software of the XGBoost prediction model (the link is: https://alanwu.shinyapps.io/ML-depression/) (Supplementary Figure 3). In this software, researchers can set the parameters of XGBoost to get the best model. At the same time, in the prediction interface, we placed 10 inputs of environmental heavy metals, so as to predict the outcome of depression.

## Discussion

In this study, we further confirmed the effects of Cd, Hg, and EtHg on depression, and these findings might help promote depression high-risk areas of people to avoid contact with hazardous metals. In practice, the government, industry, and factories involved in hazardous metals should pay close attention to heavy metal discharges, particularly in areas near residences. Reasonable plant site selection, harmless processing of heavy metal pollutants, and regular physical examination of nearby residents are imperative (15).

Through the Pearson correlation matrix, we could find that there was a strong correlation between blood Hg and MeHg. Since many studies supported the correlation between Hg and depression (6, 15–17), we exclude MeHg. Poisson regression analysis showed that the relationship between three metal elements and depression was significant: blood Cd (estimate = 0.22, *P* = 0.00000941), blood EtHg (estimate = 3.43, *P* = 0.003216), and blood Hg (estimate = −0.15, *P* = 0.001524), in which Cd and EtHg were positively correlated with depression, while Hg was negatively related with depression. This result was also consistent with other studies (18, 19). Cd induced neurotoxicity in many ways, including interfering with the blood-brain barrier, increasing oxidative stress, interfering with zinc and calcium-dependent processes and metallothionein, and inducing apoptosis. The binding of cadmium ions to mitochondria could inhibit respiration and oxidative phosphorylation. In addition, reducing cadmium absorption and blocking voltage-gated calcium (2 +) through sodium (+) dependent glutamate were associated with synaptic dysfunction. Mercury was generally considered to be a factor aggravating depression (16, 17). Contrary to expectations, the results of this study showed that mercury was a protective factor for depression. Some studies suggested that the negative correlation between mercury and depressive symptoms could be explained by the protective effect of eating fish, which might exceed any potential negative impact of mercury on the prevalence of depression (15, 18). Moreover, it was reported that ethyl mercury compounds and thiomersal easily cross the blood-brain barrier. In most cases, EtHg was transformed into highly toxic inorganic mercury compounds that significantly and persistently bind to brain tissue and cause negligible degenerative changes in nerve cells in the hypothalamic nucleus (20).

Since machine learning has advantages including easily identifying trends and patterns, no human intervention is needed, continuous improvement, and wide applications. After identifying the risk factors by Poisson regression, we fitted the machine learning model and adopted eight algorithms. It could be seen from the comparison plot that the comprehensive performance of XGBoost was better than the other seven algorithms. Then, after XGBoost calculation, the variable importance diagram is made. It can be seen that Cd, Hg, and Pb were the three most important metal elements, while Cr came last. We described the effects of Cd, and Hg on depression in the previous paragraph. It was reported that lead affects many known pathways that play a role in the development of depression, including neurogenesis and apoptosis, oxidative stress, glutathione, glutamate, calcium, and calmodulin, acetylcholine, and other neurotransmitters (15). Based on the rationality of the data, we tested the ROC curve and AUC value of the model. It could be seen that the performance of the model is good, and the AUC value reaches 0.88. Then, we used the 20% original data for prediction, and compared it with the actual value to form a confusion matrix. The accuracy of the whole model was 0.89 with 95%CI (0.87, 0.92) and the kappa value was 0.006. Based on this result, heavy metals could predict depression to a certain extent.

To make the model fitting process more intuitive, we also developed an online XGBoost prediction model application. In this model, users could change the model by adjusting 10 parameters to get a better model. Depression could also be predicted after model training. After entering the values of 10 metal elements, we could obtain the result of depression. The application could be used for related teaching and further research. These findings also contributed to the corresponding interventions for the prevention and control of depression. For example, depression might result in behavioral changes (i.e., smoking) and increased cadmium and lead exposure (19). Continuous efforts to reduce Cd through smoking cessation programs might reduce the prevalence of depression. Besides, Cr could not only improve the sensitivity of cells to insulin but also improve the function of serotonin and dopamine. It was very helpful and effective in the treatment of neurobehavioral processes such as depression and comorbidity (21).

However, this study still had some limitations. The most important factor was the cross-sectional nature of the NHANES dataset. Considering its cross-sectional design, we could not infer causality. Second, considering the large number of potential metal elements, we could not evaluate all relevant factors. Third, due to the limited types of metal elements reported in other years and the limited sample size of 10 metal elements, only 3,247 people from NHANES from 2017 to 2018 were selected for this study. Fourth, this study only analyzed the heavy metal's effect on depression, and other risk factors were not analyzed together. This was because of the limitation of data, and we could research including more risk factors in the future.

In conclusion, in Poisson regression and XGBoost models, this study further confirmed the effects of Cd, Hg, and EtHg on depression. At the same time, we also creatively made it possible for researchers to try the online prediction application to do further analysis.

## Data availability statement

The original contributions presented in the study are included in the article/Supplementary material, further inquiries can be directed to the corresponding author.

## Author contributions

JW wrote the manuscript and analyzed the data in R language. QL and XL collected the data and screened it. FX designed and reviewed the research. All authors contributed to the article and approved the submitted version.

## Conflict of interest

The authors declare that the research was conducted in the absence of any commercial or financial relationships that could be construed as a potential conflict of interest.

## Publisher's note

All claims expressed in this article are solely those of the authors and do not necessarily represent those of their affiliated organizations, or those of the publisher, the editors and the reviewers. Any product that may be evaluated in this article, or claim that may be made by its manufacturer, is not guaranteed or endorsed by the publisher.

## Supplementary material

Supplementary Figure 1 Variable importance plot (VIP) of 9 metal elements included in the XGBoost model.

Supplemental Figure 2 Receiver operating characteristic (ROC) curve of the XGBoost model with an area under curve (AUC) 0.88.

Supplementary Figure 3 **(A)** Interactive panel of online XGBoost model fitting. This panel contains hyperparameter input, accuracy plot, and VIP plot. **(B)** Interactive panel of online XGBoost model prediction. The predicted value “No” means the sample will not have depression based on the metal inputs. If the predicted value is “Yes”, the sample will have a probability of depression based on heavy metals.

## Abbreviations

WHO: World Health Organization
NHANES: National Health and Nutrition Examination Survey
BMI: body mass index
PIR: ratio of family income to poverty
OR: odds ratio
CI: confidence interval
AUC: Area under the curve
ROC: Receiver Operating Characteristic
RF: Random Forest
SVM: Support vector machine
DT: Decision Tree
BT: Boosted Tree
MARS: Multivariate Adaptive Regression Splines
KNN: K-nearest neighbors
ANN: Artificial neural network
PDQ-9: a nine-item depression screening instrument
Pb: lead
Hg: mercury
Cd: cadmium
Mn: manganese
Se: selenium
Cr: chromium
Co: cobalt
InHg: inorganic mercury
MeHg: methylmercury
EtHg: ethyl mercury.
