# Supplementary figures and images for: Machine learning model for depression based on heavy metals among aging people: A study with National Health and Nutrition Examination Survey 2017–2018
Source: Front Public Health. 2022 Aug 4;10:939758. doi: 10.3389/fpubh.2022.939758 (PMC9386350; doi:10.3389/fpubh.2022.939758)

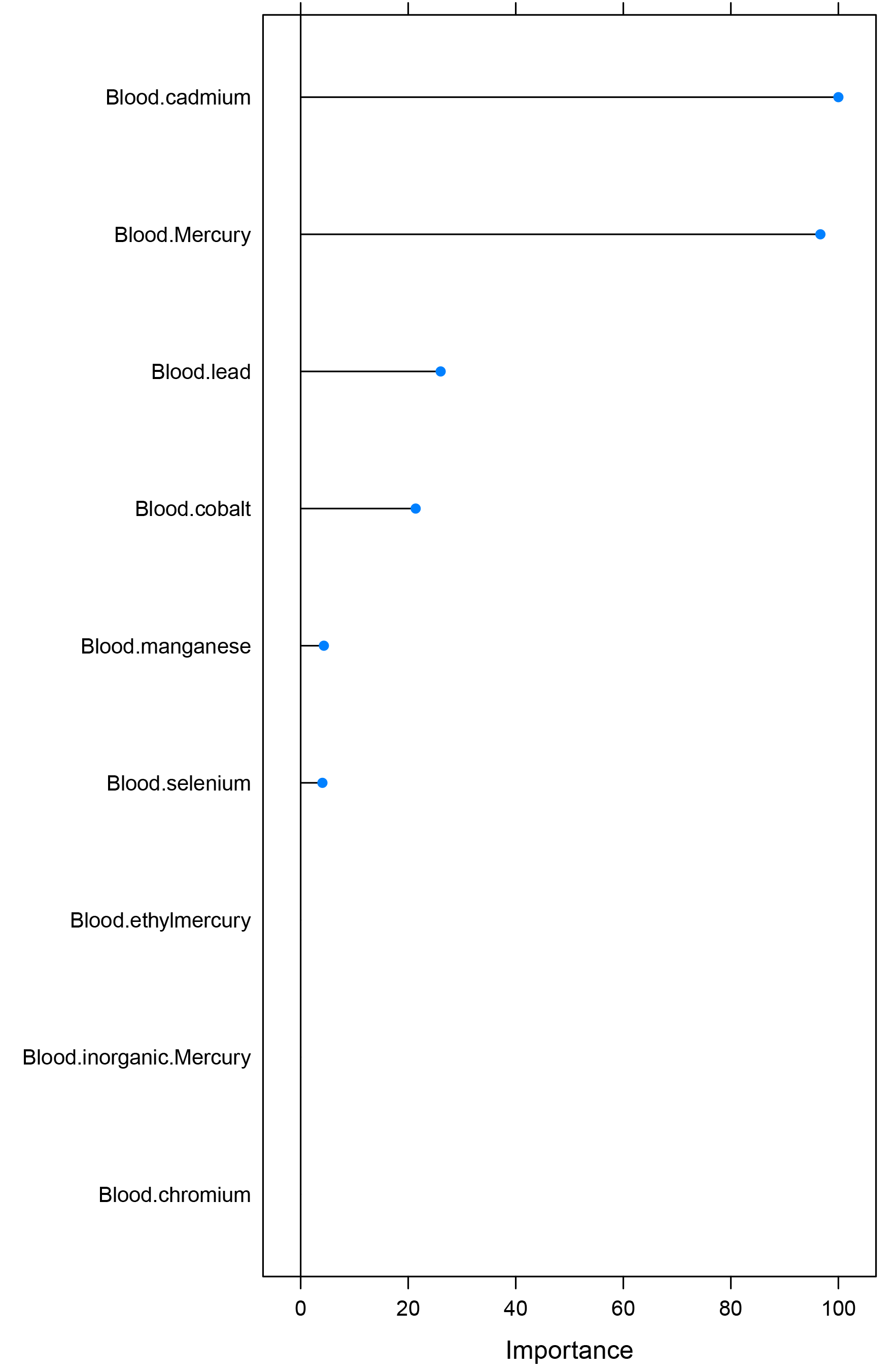

Supplement: Supplementary Figure 1 — Variable importance plot (VIP) of 9 metal elements included in the XGBoost model. [file Image_1.TIF]

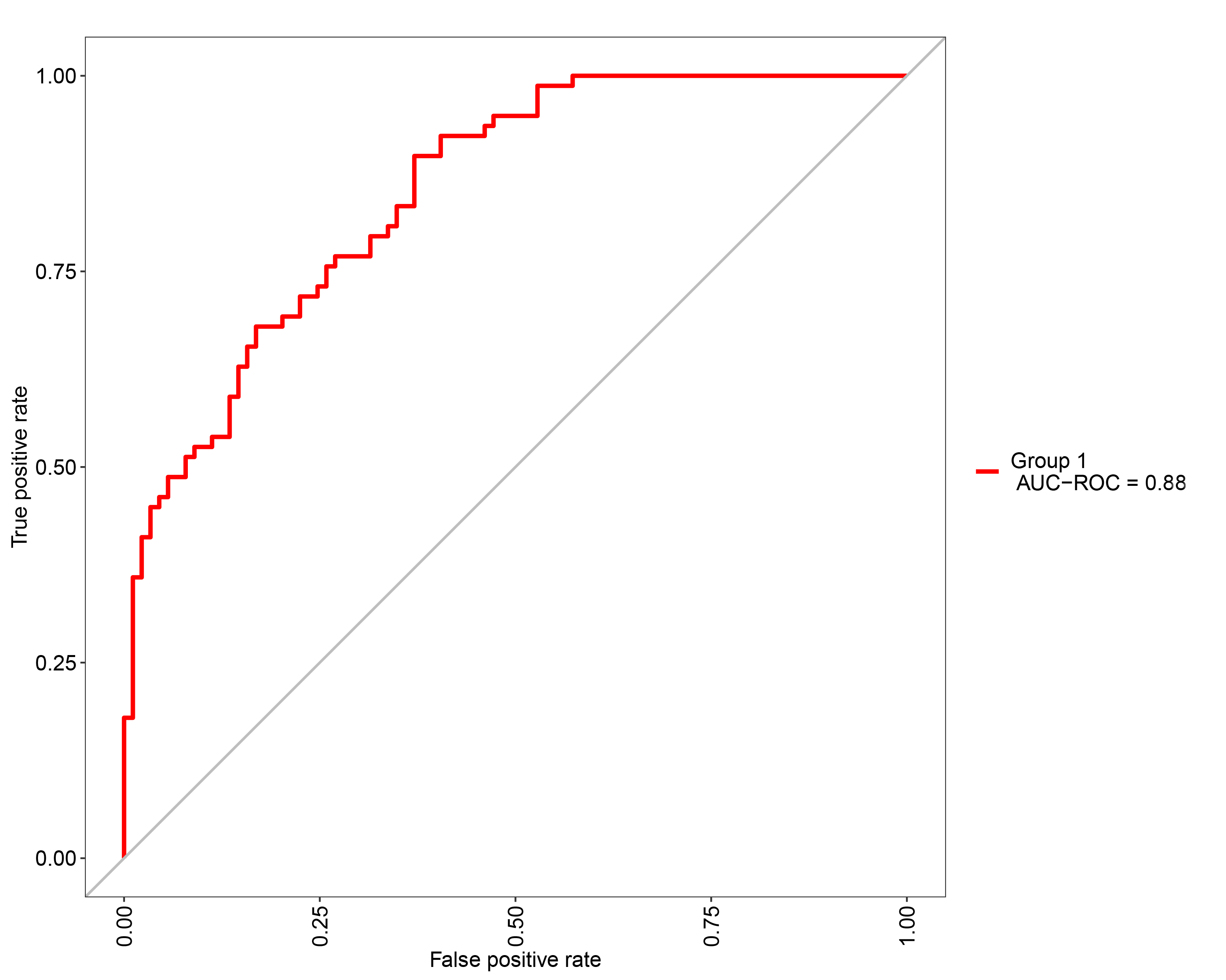

Supplement: Supplemental Figure 2 — Receiver operating characteristic (ROC) curve of the XGBoost model with an area under curve (AUC) 0.88. [file Image_2.TIF]

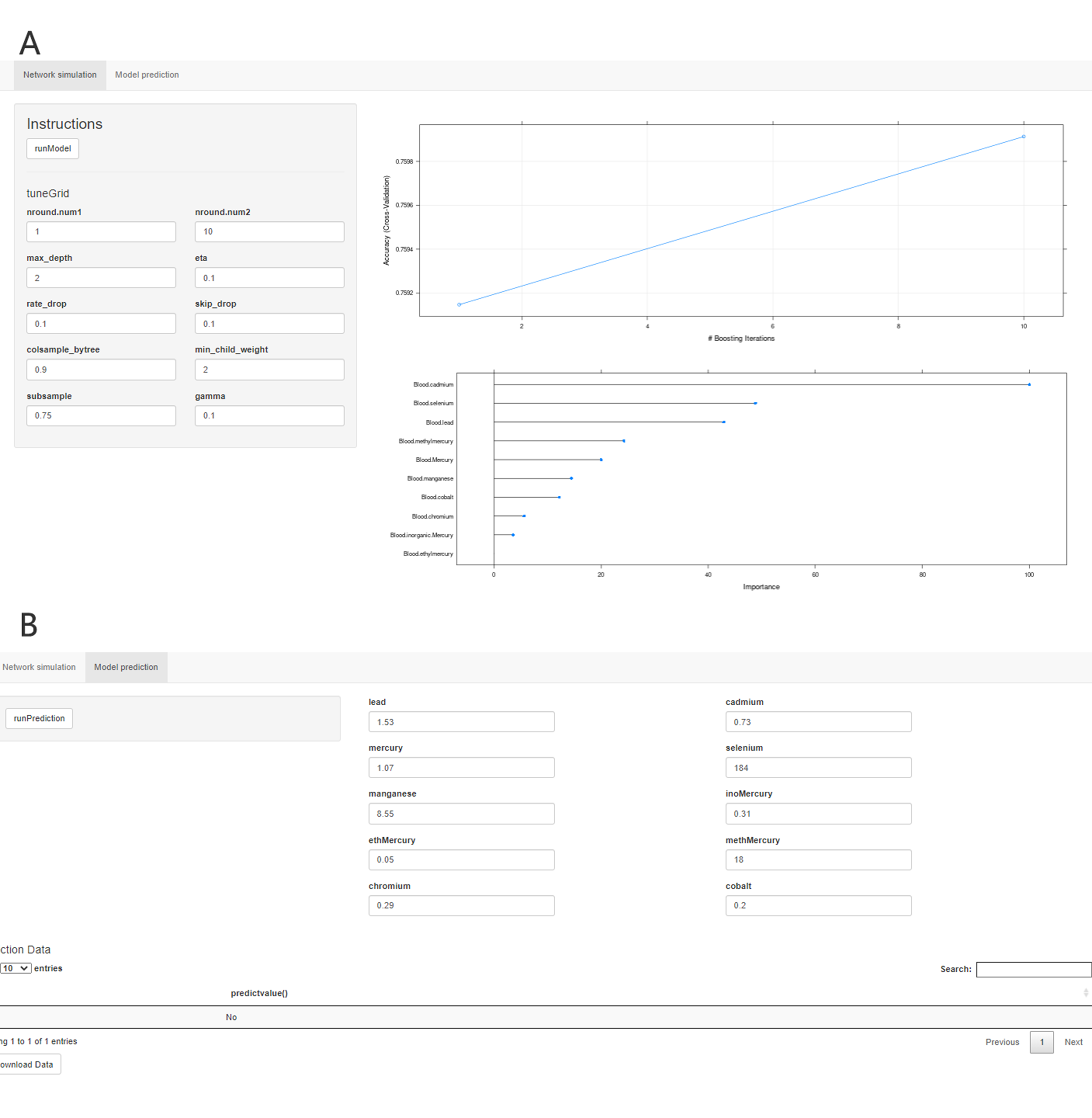

Supplement: Supplementary Figure 3 — (A) Interactive panel of online XGBoost model fitting. This panel contains hyperparameter input, accuracy plot, and VIP plot. (B) Interactive panel of online XGBoost model prediction. The predicted value “No” means the sample will not have depression based on the metal inputs. If the predicted value is “Yes”, the sample will have a probability of depression based on heavy metals. [file Image_3.TIF]
